# Assembling a cellulase cocktail and a cellodextrin transporter into a yeast host for CBP ethanol production

**DOI:** 10.1186/1754-6834-6-19

**Published:** 2013-02-04

**Authors:** Jui-Jen Chang, Feng-Ju Ho, Cheng-Yu Ho, Yueh-Chin Wu, Yu-Han Hou, Chieh-Chen Huang, Ming-Che Shih, Wen-Hsiung Li

**Affiliations:** 1 Biodiversity Research Center, Academia Sinica, 115, Taipei, Taiwan; 2 Genomics Research Center, Academia Sinica, 115, Taipei, Taiwan; 3 Department of Life Sciences, National Chung Hsing University, 402, Taichung, Taiwan; 4 Agricultural Biotechnology Research, Center, Academia Sinica, 115, Taipei, Taiwan; 5 Department of Ecology and Evolution, University of Chicago, 60637, Chicago, IL, USA

**Keywords:** Cellulosic ethanol, Crystalline cellulose, Cocktail formulation, Synthetic biology, Consolidated bioprocess

## Abstract

**Background:**

Many microorganisms possess enzymes that can efficiently degrade lignocellulosic materials, but do not have the capability to produce a large amount of ethanol. Thus, attempts have been made to transform such enzymes into fermentative microbes to serve as hosts for ethanol production. However, an efficient host for a consolidated bioprocess (CBP) remains to be found. For this purpose, a synthetic biology technique that can transform multiple genes into a genome is instrumental. Moreover, a strategy to select cellulases that interact synergistically is needed.

**Results:**

To engineer a yeast for CBP bio-ethanol production, a synthetic biology technique, called “promoter-based gene assembly and simultaneous overexpression” (PGASO), that can simultaneously transform and express multiple genes in a kefir yeast, *Kluyveromyces marxianus* KY3, was recently developed. To formulate an efficient cellulase cocktail, a filter-paper-activity assay for selecting heterologous cellulolytic enzymes was established in this study and used to select five cellulase genes, including two cellobiohydrolases, two endo-β-1,4-glucanases and one beta-glucosidase genes from different fungi. In addition, a fungal cellodextrin transporter gene was chosen to transport cellodextrin into the cytoplasm. These six genes plus a selection marker gene were one-step assembled into the KY3 genome using PGASO. Our experimental data showed that the recombinant strain KR7 could express the five heterologous cellulase genes and that KR7 could convert crystalline cellulose into ethanol.

**Conclusion:**

Seven heterologous genes, including five cellulases, a cellodextrin transporter and a selection marker, were simultaneously transformed into the KY3 genome to derive a new strain, KR7, which could directly convert cellulose to ethanol. The present study demonstrates the potential of our strategy of combining a cocktail formulation protocol and a synthetic biology technique to develop a designer yeast host.

## Background

To achieve an economical cellulosic biofuel production, three conditions should be met, namely an efficient process, a good host, and a good cellulolytic enzyme system. Compared to other bioprocesses such as SHF (separate hydrolysis and fermentation) and SSF (simultaneous saccharification and fermentation) [1], CBP (consolidated bioprocessing) is considered to have the potential to be more economical in the use of enzymes and in operational cost [2]. To achieve CBP, a host that can carry out both lignocellulose hydrolysis and ethanol fermentation is required. Although several microbes, including *Saccharomyces cerevisiae, Kluvyveromyces marxianus, Zygosaccharomyces bailii* and *Zymomonas mobilis*, have been considered good ethanol producers from sugars, their genomes lack genes for cellulolytic enzymes [3-6].

The lignocellulose contains the insoluble lignin/hemicellulose microfibrils and the crystalline cellulose buried within the architecture. Thus, to digest lignocelluloses, many fungi secrete a cellulolytic enzyme cocktail. For example, the brown-rot fungus *Trichoderma reesei* simultaneously expresses three kinds of endo-β-1,4-glucanases (EG) and two kinds of cellobiohydrolases (CBH) when treated with a barley straw substrate [7]. The rumen fungus *Neocallimastix patriciarum* W5 is able to grow on rice straw powder, by expressing an enzyme cocktail with highly efficient β-glucosidases (BGL) [8,9]. *Aspergillus niger* can express EG and BGL enzymes, and its BGL has been purified and manufactured as the commercial Novozyme 188 [10,11]. Moreover, the model cellulolytic *Neurospora crassa* has seven major facilitator superfamily (MFS) sugar transporters, and the cellodextrin transportors (CDT-1 and CDT-2) have been shown to facilitate transport of cellobiose, cellotriose, and cellotetraose into the cytoplasm [12,13]. But, these cellulolytic consumers are not suitable for the fermentation industry, because of a low growth rate, the requirement of a specific culture medium, the requirement of a special inducing condition, or the inability to produce a serviceable biofuel product [14]. However, these cellulolytic enzyme systems may be used to engineer a yeast host for the cellulosic ethanol industry.

In principle, cellulosic polysaccharides can be hydrolyzed when three types of cellulase (EG, CBH, and BGL) are used [15-17]. However, a cellulolytic fungus usually possesses different cellulases of the same type, suggesting that multiple enzymes are more efficient than single enzyme. Indeed, the commercial Celluclast 1.5 L, which contains mainly EG and CBH activities, achieves a synergism with the mixture of CBHI, CBHII, EGI, and EGII of *T. reesei*[7]. The synergistic effect of different cellulases on lignocellulosic substrate hydrolysis, such as cellulases from *Penicillium echinulatum* and *T. reesei,* is known in SSF applications [18,19]. The commercial Novozyme 188 is one of the most commonly used enzyme for promotion of a synergistic effect with *Trichoderma* cellulases, and a cocktail formulation supplemented with this BGL can efficiently produce fermentable glucose [11,20,21]. Although a concept of designing a highly efficient cellulase mixture for hydrolysis of cellulose has been reported [21], a designer host for highly efficient cellulase cocktail production has not yet been achieved.

To achieve CBP, research has been pursued in a number of host organisms including *Escherichia coli*, *Bacillus subtilis*, and especially *S. cerevisiae*[2]. Several different approaches have been applied to select or engineer fermentable yeasts for cellulosic material utilization, such as screening a yeast for utilizing cellobiose [22], or for transformation of cellulase genes [23,24], high level secretion of cellobiohydrolases [25], and transformation of polysaccharide transporter genes [13]. However, a good CBP host remains to be found or constructed [2]. Recently*, K. marxianus* was proposed as a potential yeast host to develop a CBP for converting cellulosic materials into bioethanol [4,24,26]. We have recently isolated a kefir yeast, *K. marxianus* KY3, which is efficient in fermenting ethanol from hexose and pentose sugars and has many other advantages, including thermo-tolerance, a high growth rate, broad growth temperature and pH ranges, efficient secretion of heterologously expressed proteins and a broad substrate spectrum [23]. Moreover, a synthetic biology tool, called the promoter-based gene assembly and simultaneous overexpression (PGASO) technique, was developed and used to insert a five-gene cassette, including three cellulase genes (CBH, EG and BGL), in a predesigned order into the genome of KY3, resulting in a recombinant KR5 strain [24]. KR5 could directly convert beta-glycan to ethanol, but could not utilize crystal avicel, which has a more compact cellulose structure than beta-glycan.

**Figure 1:**
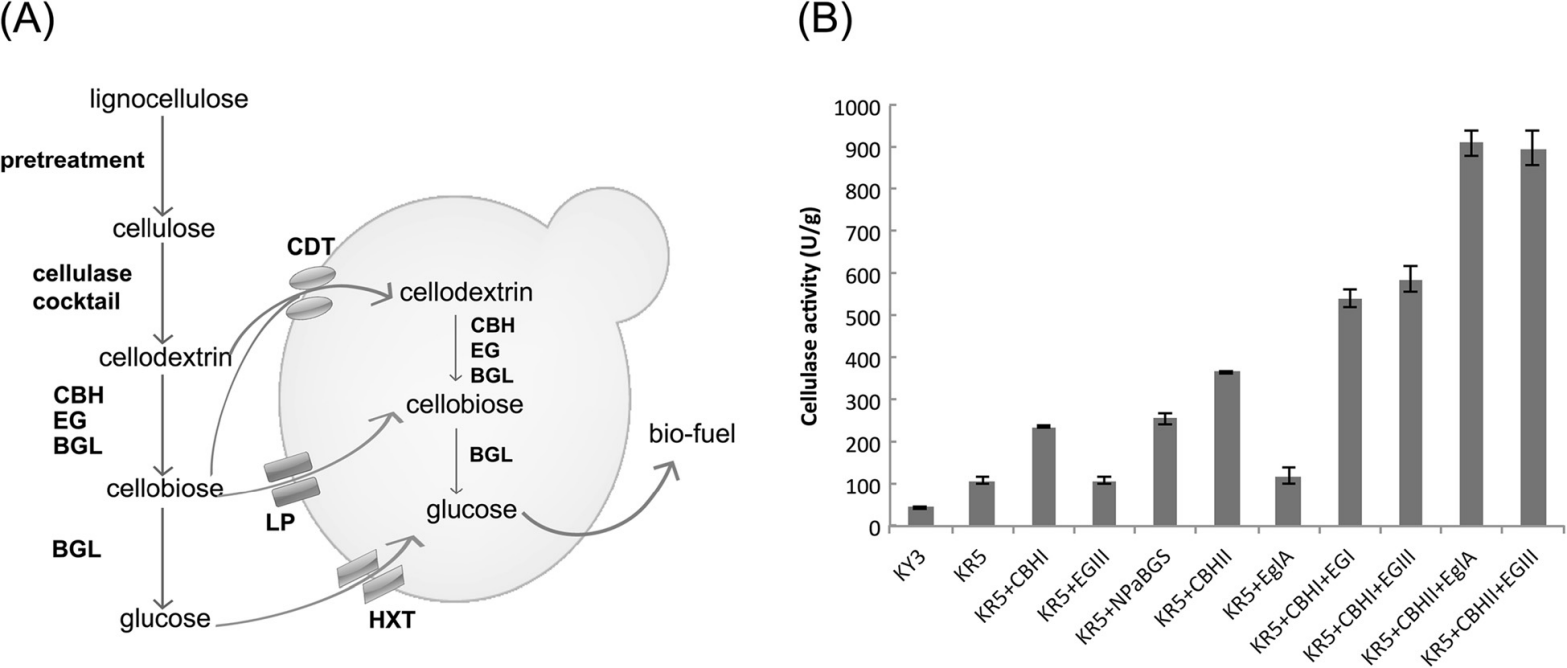
**The cellulose utilization pathways and cellulase cocktail formulation.** (**A**) The fungal cellulose utilization systems in yeast during simultaneous saccharification and fermentation of cellulose. (**B**) The filter paper activity (FPA) assay of potential enzyme synergistic effects with the crude enzyme of KR5. Several different commercial cellulases, including EG (EglA and EGIII), CBH (CBHI and CBHII) and BGL (purified NpaBGS), were used for cocktails with the supernatants of KR5 by mixing equivalent proportions of volume individually. One unit of FPA is defined as one μmol reducing sugar released from filter paper in one minute. The cellodextrin transport pathway includes a cellodextrin transporter (CDT) and intracellular cellulases (EG, CBH, and BGL). The sugar catabolism pathway present in yeasts includes hexose transporters (HXT) and lactose permease (LP). In SSF, pretreatment and extracellular cellulase cocktail process may both be used.

Since a CBP host should possess the capability for simultaneous saccharification and fermentation, KR5 needs to be improved. For this purpose, we need to know which cellulase genes to transform into the host genome because it is difficult to transform many genes into a genome. In this study, we have developed an approach to select genes to produce an efficient cellulase cocktail. Using this strategy, we have chosen five cellulase genes from previous studies to form an efficient cocktail. In addition, we also chose a cellodextrin transportor gene. We then transformed these six genes and a selection marker gene into the KY3 genome using PGASO. The resulting strain, KR7, was tested for ethanol conversion from the crystalline substrate, avicel, and the cellulose consumption pathway is shown in Figure 1A. Our study shows that expressing enzymes by our method in *K. marxianus* KY3 has the potential to create CBP strains.

## Results

### An enzyme cocktail formulation assay for selecting synergistic enzymes

In our previous study, we have engineered a recombinant *K. marxianus* KY3 strain, called KR5, that possesses the *cbhI*, *egIII*, and *npabgs* genes and can secrete the CBH, EG and BGL enzymes simultaneously [24]. Although KR5 can grow on media with cellodextrins, such as cellobiose and beta-glycan, it does not have the ability to utilize more complex cellulose substrates, such as filter paper and avicel. As FPA (the filter paper activity) is a widely accepted assay for the overall cellulase activity, we conducted an FPA assay, using the supernatant of the KR5 culture as the source of crude enzymes. The FPA data indicated only a 110 U/g overall cellulase activity in KR5 (Figure 1B).

To improve the enzyme activity of KR5, we developed an enzyme cocktail strategy to investigate the synergistic actions with the crude enzymes of KR5. Many different glycosyl hydrolase (GH) family cellulases, including EglA (GH5), EGIII (GH5), CBHI (GH7), CBHII (GH6), and purified NpaBGS (GH3), were individually added to the cocktail with the crude enzymes of KR5 by mixing equivalent proportions of volume. The enzyme cocktail results showed that increasing the amounts of CBHI and NpaBGS only slightly improved the total cellulolytic ability of KR5, while the addition of CBHII resulted in a significant increase in the total FPA activity (Figure 1B). In terms of cellulase activity (U/g), we note from Figure 1B: (1) KR5+CBHII+EglA is more than twice of either KR5+CBHII or KR5+EglA, (2) KR5+CBHII+EglA is higher than the sum of KR5+CBHII and KR5+EglA, (3) KR5+CBHII+EGIII is more than twice of KR5+CBHII or KR5+EGIII, and (4) KR5+CBHII+EGIII is higher than the sum of KR5+CBHII and KR5+EGIII. These observations suggest synergism between CBHII and EglA, between CBHII and EGII or among the five cellulases.

### Improving the cellodextrin consuming ability of the host

**Figure 2:**
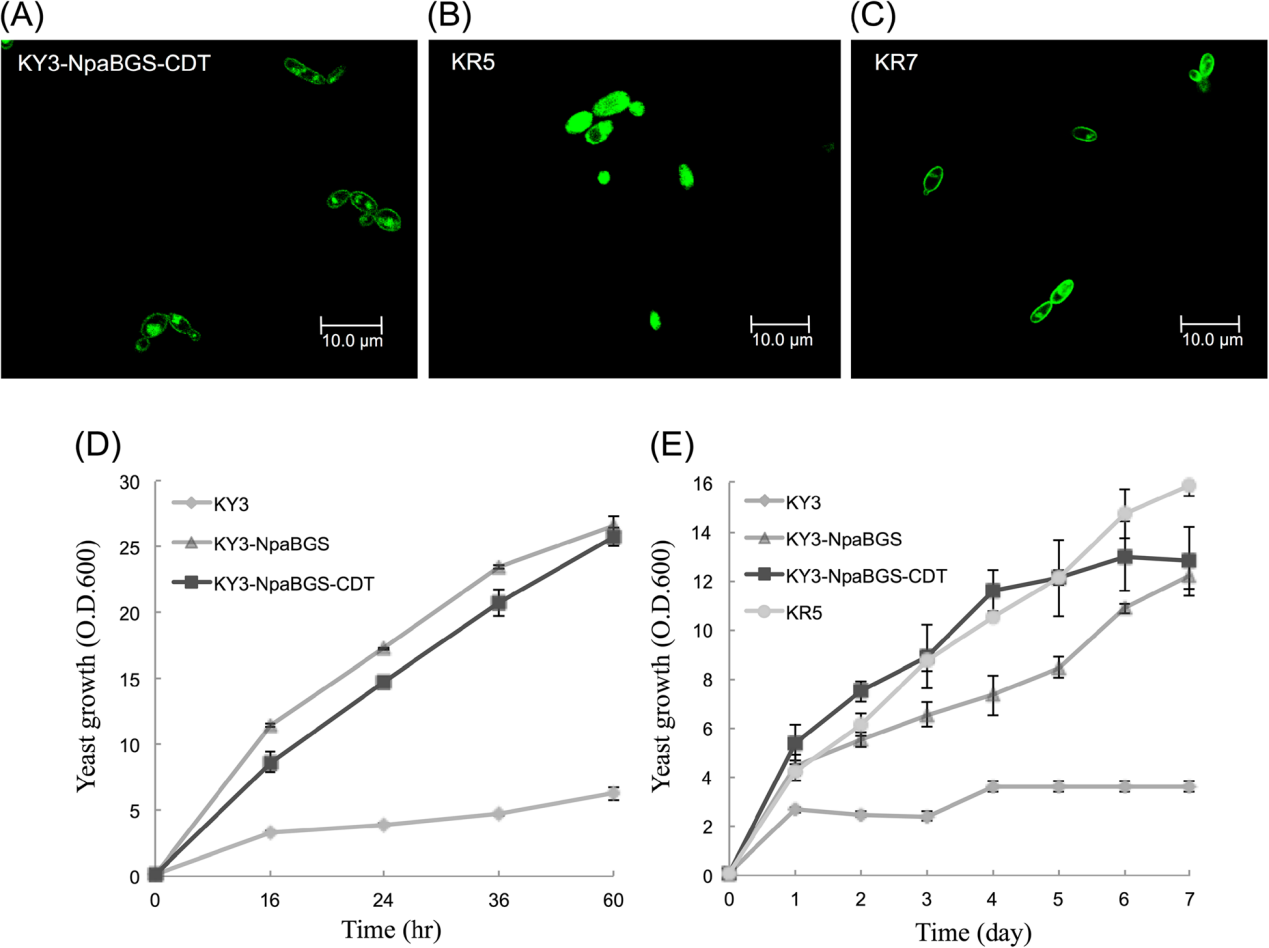
**The growth curve and fluorescence microscopy photograph of the engineered yeast strains.** (**A**) Three recombinant strains KY3-NpaBGS-CDT, KR5 and KR7 that expressed green fluorescent protein in cytoplasm. KR5 was employed as a control. (**B**) and (**C**) the CDT gene fusion with GFP gene was expressed on the cell membrane of KY3-NpaBGS-CDT and KR7, respectively. The growth curve was conducted by the engineered yeast strains using cellobiose (**D**) and cellodextrin (**E**) as the solo substrate, respectively.

In our previous studies, we showed that *K. marxianus* KY3 transformed with the NpaBGS gene (called KY3-NpaBGS) secreted BGL and improved cellobiose consumption [9,23]. However, KY3-NpaBGS did not show a good efficiency in utilizing more complex substrates, such as cellotriose, cellotetraose and cellodextrins. Also, one of the cellodextrin transporters of *N. crassa*, CDT-1, has been engineered in the *S. cerevisiae*, and the transformation improved the cellodextrin consuming ability in the yeast host [13]. We therefore decided to test whether expression of a cellodextrin transporter gene will improve the cellodextrins consumption ability of KY3-NpaBGS. A gene encoding the membrane cellodextrin transport (CDT-1) was amplified from the cDNA of *N. crassa* and fused with a green fluorescent protein (GFP) gene. The gene cassette, *cdtI-gfp,* was cloned in the pKLac2-kan vector and co-transformed into KY3-NpaBGS. The resultant strain KY3-NpaBGS-CDT, which showed *cdtI-gfp* on the cell membrane, was confirmed by fluorescence microscopy (Figure 2A). The strain KR5 that expresses the green fluorescent protein in cytoplasm was employed as a control (Figure 2B). We compared the growth rates of these KY strains on different carbon sources. When glucose was used as the carbon source, all the experimental strains, including KY3, KR5, KY3-NpaBGS-CDT and KY3-NpaBGS, showed an equal growth rate (data not shown). When cellobiose was used as the carbon source, both KY3-NpaBGS-CDT and KY3-NpaBGS grew faster than KY3, while KY3-NpaBGS-CDT grew at a slightly slower rate than KY3-NpaBGS (Figure 2D). On the other hand, when a more complex cellodextrin was used as the carbon source, KY3-NpaBGS-CDT grew significantly faster than KY3-NpaBGS and KY3 (Figure 2E). Moreover, KR5, which possessed two more kinds of cellulase, also grew significantly faster than KY3-NpaBGS and KY3 (Figure 2E). These results suggest that both substrate transport and cellulose digestion are two limiting factors for yeast to utilize cellodextrin for its growth.

### A CBP strain construction

**Figure 3:**
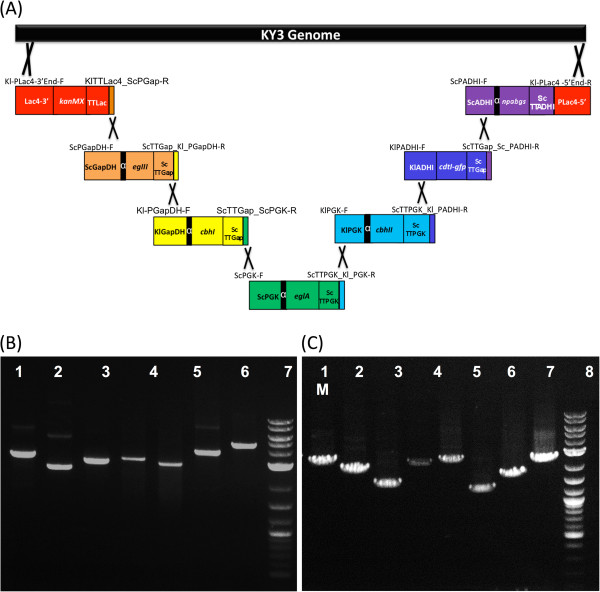
**Genomic integration of seven gene cassettes in KR7.** Each of the seven gene cassettes contains an independent promoter, the alpha factor, a gene coding region, a terminator, and a 55 bp fragment homologous to its neighboring cassette. (**A**) The gene cassettes assembled in the predesignated order, *kanMX, egIII*, *cbhI*, *eglA*, *cbhII*, *cdtI-gfp,* and *npabgs*, and are shown in red, orange, yellow, green, blue, dark blue, and purple, respectively. (**B**) The seven gene cassettes were amplified by PCR for transformation with seven primer pairs and the PCR products resulted in seven specific amplicons: 1: *kanMX,* 2: *egIII*, 3: *cbhI*, 4: *eglA*, 5: *cbhII*, 6: *cdtI-gfp,* and 7: *npabgs.* (**C**) The order of the gene cassettes was confirmed by PCR with seven internal primer pairs and the PCR products resulted in eight specific amplicons: 1: Lac4-*kanMX,* 2: *kanMX-egIII*, 3: *egIII-cbhI*, 4: *cbhI-eglA*, 5: *eglA-cbhII*, 6: *cbhII-cdtI-gfp,* 7: *cdtI-gfp-npabgs,* and 8: *npabgs-*Lac4.

In the above, the enzyme cocktail formulation assay indicated that CBHII plus either EglA or EGIII could work with synergism to improve the cellulolytic efficiency of the crude enzymes of KR5, which possessed the *cbhI*, *egIII* and *npabgs* genes. To construct a CBP host, we therefore chose the five cellulase genes of *cbhII* (GH6, 1,4-beta-D-glucan cellobiohydrolase II from *T. reesei*), *cbhI* (GH7, cellobiohydrolase I from *T. reesei*), *egIII* (GH5, endoglucanase III from *T.reesei*), *eglA* (GH5, endoglucanase A from *A. niger*) and *npabgs* (GH3, beta-glucosidase from *Neo. patriciarum*) and aslo the cellodextrin transporter gene with gfp and transformed these six genes and the selection marker gene, *kanMX,* into the *K. marxianus* KY3 genome in a single step using PGASO. Each gene cassette contains an individual promoter sequence, a leader sequence, a coding sequence, a terminator structure sequence and a 55 bp sequence at the 3’ end of the gene cassette that is identical to the 5’ end of the adjacent downstream gene cassette. The seven gene cassettes were constructed in the predesignated order *kanMX, egIII*, *cbhI*, *eglA*, *cbhII*, *cdtI-gfp,* and *npabgs*; their promoter sequences were KlLac4, ScGapDH, KlGapDH, ScPGK, KlPGK, KlADHI, and ScADHI, respectively (Figure 3A). All of these promoters were constitutive promoters from *S. cerevisiae* (Sc) or *K. lactis* (Kl)*,* and they showed only 40-55% sequence identity between each other in their 5’ upstream regions. A portion of the 5’ end of the promoter sequence for the *kanMX* gene cassette and a portion of the 3’ end of the *npabgs* gene cassette are homologous to a Lac4 promoter site in the KY3 genome in order to facilitate site-specific insertion. The seven gene cassettes were amplified by PCR for transformation with seven primer pairs and the PCR products resulted in seven specific amplicons, respectively, with 3165 bp, 2484 bp, 2833 bp, 3052 bp, 2824 bp, 3680 bp and 4519 bp (Figure 3B). After the gene cassettes were introduced into the cells, the transformants were selected with 200 ug/ml G418 on the YPGK plates.

To verify that these gene cassettes were assembled in the correct order, eight internal primer pairs spanning the gap regions of each cassette were designed [see Additional file 1]. The PCR products showed seven specific bands with correct sizes of 2400 bp, 2293 bp, 1712 bp, 2513 bp, 2980 bp, 1422 bp, 1885 bp, and 2575 bp, respectively (Figure 3C). The resultant strain was named KR7, which was integrated with a DNA fragment of 22,557 bp. The transformation accuracy of gene cassettes assembly was workable, as on average 3% colonies were found to have the correct assembly with the predesigned order.

**Figure 4:**
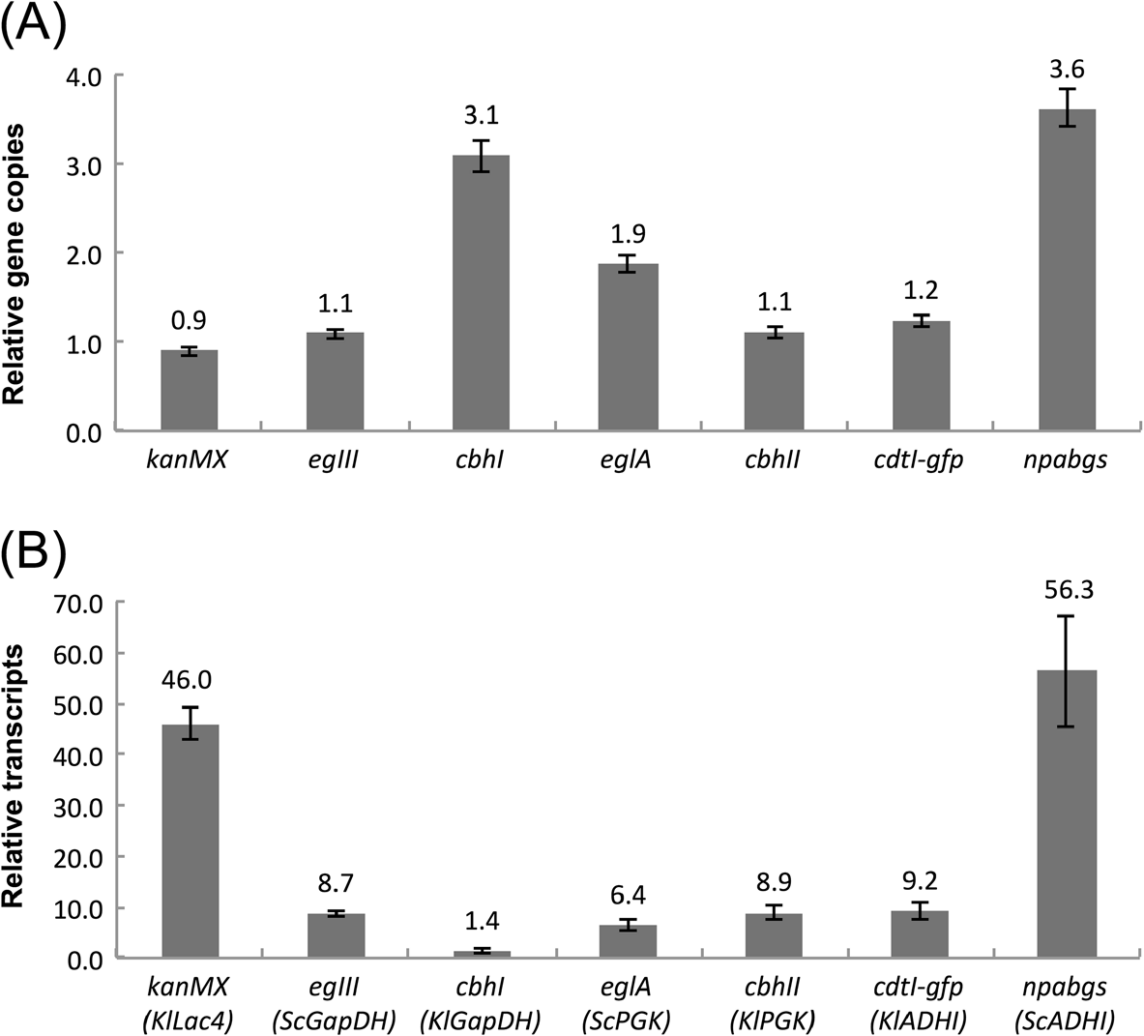
**Quantitative PCR analysis of the seven gene cassettes in KR7.** (**A**) Relative copy numbers of inserted genes. (**B**) Relative mRNA levels of cellulase genes inserted in KR7. The relative ratios of the seven genes and their transcripts are shown in comparison to the endogenous alg9 gene in KR7 at 40°C in YPAD culture. The promoter names of the individual genes were given in the brackets.

To determine the inserted copy numbers of the seven genes, genomic DNA was isolated from KR7 for quantitative PCR analysis, which was performed with seven gene-specific primer sets using the UPL system [see Additional file 1]. The observed inserted copy-number ratios of the seven genes were approximately 2 (*kanMX*) : 2 (*egIII*) : 6 (*cbhI*) : 4 (*eglA*) : 2 (*cbhII*) : 2 (*cdtI-gfp*) : 7 (*npabgs*) relative to the indigenous alg9 gene (2 copies) in KR7 (Figure 4A). For monitoring the multiple genes expression system, total RNA was also isolated from KR7 that was grown at 30°C in YPD for quantitative PCR analysis, and alg9 was employed as the reference gene. The mRNA levels of the seven genes were 46.0 (*kanMX*) : 8.7 (*egIII*) : 1.4 (*cbhI*) : 6.4 (*eglA*) : 8.9 (*cbhII*) : 9.2 (*cdtI-gfp*) : 53.6 (*npabgs*) relative to the indigenous alg9 gene in KR7 (Figure 4B). The data suggested that the KlGapDH and ScPGK promoters, which drove *cbhI* and *eglA*, respectively, might be inherently weaker than the other constitutive promoters in KR7 under the condition used.

### Characterization of the secreted cellulases of the CBP strain

**Figure 5:**
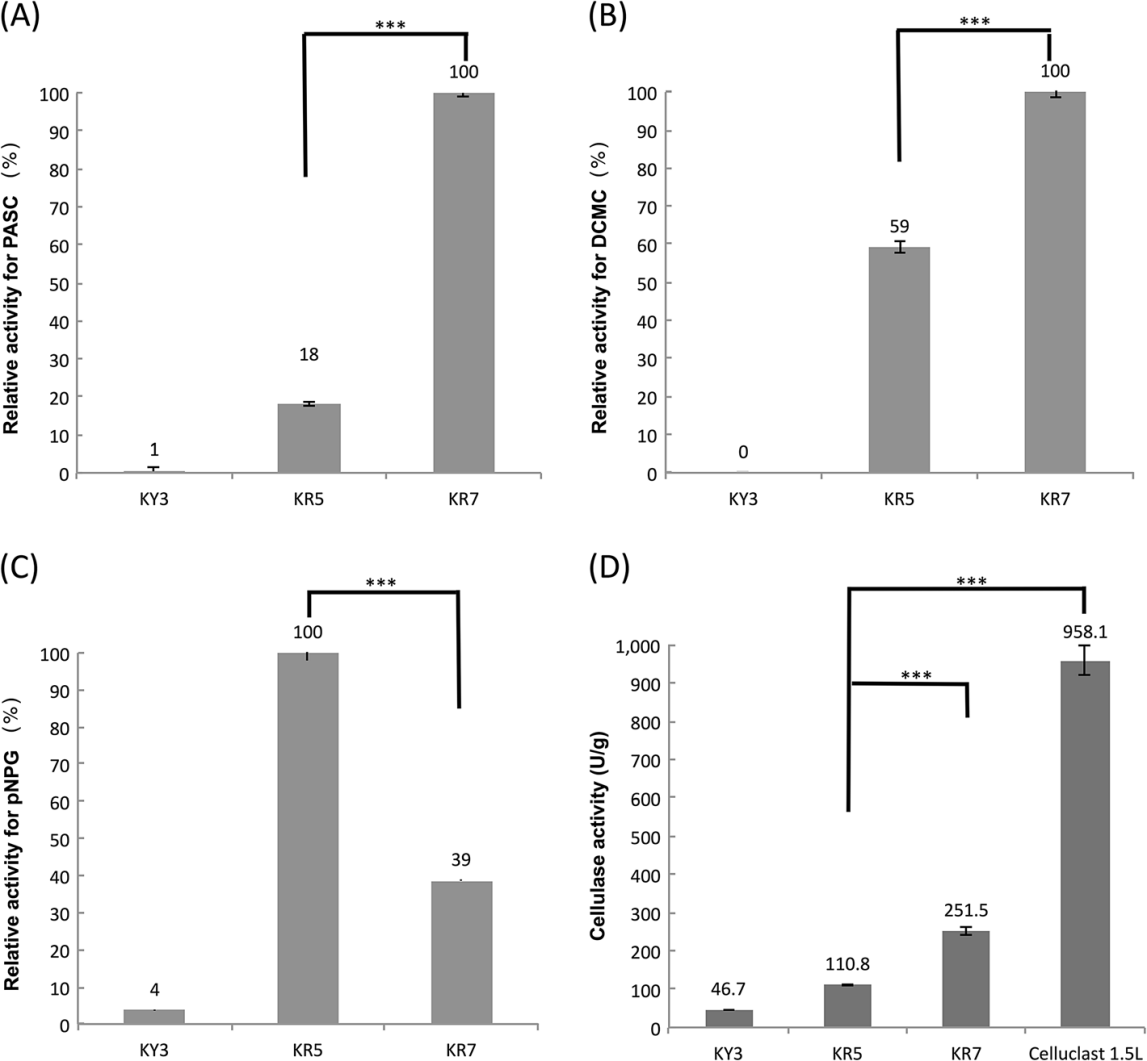
**Cellulolytic enzyme assays of engineered yeasts.** (**A**) CBH relative activity with PASC as the sole substrate. (**B**) EG relative activity with CMC as the sole substrate. (**C**) BGL relative activity assay with pNPG as the sole substrate. (**D**) Total specific cellulase activity assay with filter paper as the sole carbon substrate. The samples collected from KY3, KR5 and KR7 cultures were estimated using the same protein concentration for enzyme reaction at 40°C. One unit of FPA is defined as one mmol reducing sugar releasing from filter paper in one minute. *: P < 0.05 (significant), **: P < 0.01; ***: P < 0.001; N.S., non-significant.

To quantify the activities of secreted cellulases, the supernatants of KY3, KR5 and KR7 cultures were harvested and concentrated for analysis at 40°C. The relative activity assay with Dye-CMC as the substrate suggested that the EG activity in the supernatant of KR7 was 1.68 folds higher than that of KR5, probably due to secretion of the extra EglA (Figure 5A). The CBH relative activity assay was performed with PASC (phosphoric acid-swollen cellulose) as the substrate, and the results indicated that the supernatant of KR7 had a 5-fold improvement over KR5, probably due to the presence of the extra CBHII in KR7 (Figure 5C). The relative activity assay with pNPG as the substrate showed that the BGL activity in the supernatant of KR7 was not improved, being 2.5-fold lower than KR5 (Figure 5B). The overall cellulase activity assay was performed using the filter paper as the substrate, and the supernatant of KR7 culture showed a 2.27-fold improvement in the cellulase activity over KR5 (Figure 5D). These data demonstrated the simultaneous introduction of five fungal cellulase genes into the KY3 host, and their gene products were secreted with cellulolytic enzyme activity. Although the specific cellulase activity of the supernatant of KR7 was still 3.8-fold lower than the commercial Celluclast 1.5 L, it showed that a CBP host can be constructed by our strategy (Figure 5D).

### Evaluation of cellulosic ethanol conversion

**Figure 6:**
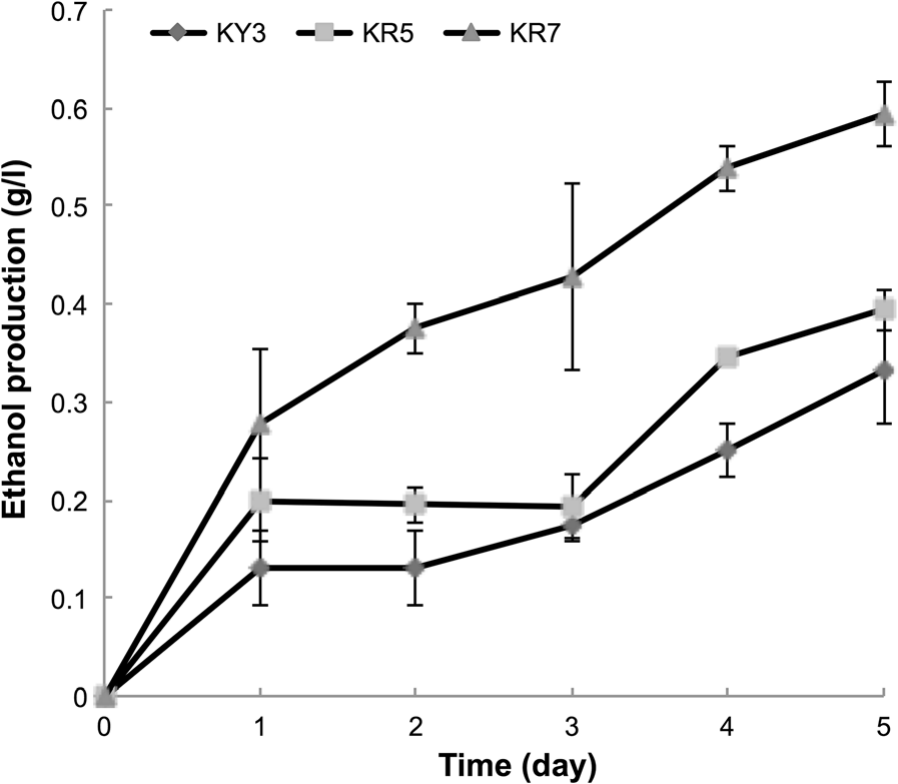
**The cellulosic ethanol production assays.** KY3, KR5, and KR7 were inoculated with OD of 20 cells, and a 10% avicel was added into 10 ml YP medium as the solo carbon source. The semi-anaerobic batch cultures were performed at 40°C with 250 rpm shaking.

For a rigorous evaluation of the cellulolytic efficiency of the KR7 strain, many parameters should be considered. In this study, however, we examined the differences between the engineered yeast strains in utilizing a crystalline substrate “avicel” for ethanol production in a simple way. To deal with the tight structure of avicel, an ultimate condition with a saturated substrate concentration, a pretreatment with high temperature sterilization, high cell density inoculum, and semi-anaerobic culturing were employed in a CBP test. Because a higher temperature is more efficient for ethanol production by *K. marxianus* KY3 [24] and for the enzymatic reaction of cellulases, the test was conducted at 40°C for ethanol production. All three strains, KY3, KR5, and KR7, were inoculated with O.D. of 20 cells, and a 10% avicel was added into 10 ml YP medium as the carbon source. As shown in Figure 6, KR7 could use avicel as the sole carbon source for ethanol conversion and produce significantly more ethanol (0.42 g/l yield) than KY3 and KR5 in cultures grown for two days. Although KR5 could also produce 0.39 g/l ethanol at the fifth day, the yield of KR7 had reached 0.6 g/l by that time. These data indicated that the cellulosic ethanol converting ability of KR7 was not only significant higher, but also 2.5-fold faster than KR5 (Figure 6). However, due to the saturated substrate level of 100 g/L avicel was over loaded, it was difficult to quantify the amount of avicel substrate that could be degraded during the fermentation of KR7.

## Discussion

Using an enzyme cocktail formulation for lignocellulosic substrate hydrolysis, such as cellulases from *P. echinulatum*, *A. niger*, and *T. reesei*, is a well-known strategy for cellulose pretreatment in SSF. The *Trichoderma* cellulase mixture is one of the most commonly used enzymes in practical applications. However, it often requires a cellulase cocktail supplemented by BGL of *Aspergillus* for promotion of a synergistic effect to produce fermentable glucose from raw biomass material [11,20,21]. To design a practical enzyme cocktail formulation, expressing a group of cellulases with suitable proportions might be efficient for a specific cellulose substrate hydrolysis. In this study, an enzyme cocktail strategy was applied to choose several synergistic cellulases from different fungi and a designer concept was achieved for a cellulase cocktail production in a single host, using a synthetic biology technique.

Presently, degradation of lignocellulose requires large quantities of cellulases to release glucose from plant cell wall [2,27]. The high cost of cellulases is the current bottleneck of biofuel industry. Moreover, the special culturing and induction condition required by a fungal host is a limiting step of traditional enzyme production technologies [7,10]. In this study, to reduce the culturing time and cost, a yeast engineering approach was used to transform cellulase genes from different fungi into the KY3 host genome. In this study, a recombinant strain KR7 with five different cellulases from *Trichoderma*, *Aspergillus*, and *Neocallimastix*, was constructed. Although the specific FPA activity of the secreted enzymes of KR7 was still lower than the commercial enzymes of Celluclast 1.5 L from *Trichoderma*, this yeast system has many advantages as a cell factory for enzyme production, such as a high growth rate, enzyme secretion, and high temperature fermentation. However, it still needs further improvement before industry application. For example, elevation of the enzyme production could be achieved by improving the enzyme secretion ability with better secretion leader sequence [28] or by reforming the protein folding with related chaperon gene co-expression [29,30]. Furthermore, the enzyme activity can be further improved by increasing the thermostability by addition of specific ions in the growth media [31].

Although KR5 possessed the *cbhI* gene of *T. reesei*, it could not efficiently hydrolyze the filter paper. There might be two possible reasons: First, as the *cbhI* gene in KR5 was regulated by the KlGapDH promoter, which was the weakest promoter we used with the PGASO method, KR5 might not have been able to express sufficient cellobiohydrolases for the filter paper hydrolysis. Second, the enzyme cocktail results showed that the addition of CBHI alone only slightly improved the cellulolytic ability of KR5, but the addition of CBHII resulted in a significant increase in the total FPA activity, probably because of the joint effect of CBHI and CBHII as KR5 already possessed the *cbhI* gene. This result is similar to a previous enzyme co-expression study [25], which suggested that the joint effect of different cellulases is important for cellulose hydrolysis. We will study this possibility in the future.

To improve the cellulose consumption ability of a host, the cellulose hydrolysis enzyme system should be able to coordinate with their cellulose hydrolyte transporting mechanisms. Although *K. marxianus* could use the lactose permease (Lac12) for cellobiose transport [32], it was not efficient for ethanol conversion. Other fungus, such as *N. crassa*, relies on a high-affinity cellodextrin transport system for rapid growth on cellulose [12]. We have shown that the yeast strain KY3-NpaBGS-CDT, which co-expressed the cellodextrin transport (CDT-1) gene of *N. crassa* and the *NpaBGS* gene of *Neo. patriciarum*, could increase the growth rate under a cellodextrin substrate. Compared with KY3-NpaBGS, which possessed a single enzyme, KY3-NpaBGS-CDT grew slightly faster in the first five days, while KR5, which expressed a combination of cellulases, could digest more cellodextrins and showed a higher growth mass in the seven-day culture. KR7 provided a better solution to assemble the two functional features via the PGASO method in a single yeast for cellulolytic ethanol production.

Our CBP ethanol production study showed co-expressing exogenous fungal genes in a yeast host can convert cellulose to ethanol. Since the avicel was pretreated with steam sterilization in the autoclave, smaller cellulose fragments, such as cellodextrins, might have been released in the culture medium. However, there was no detectable reducing sugar in this medium. Compared to KR5, KR7 expressed the *cdtI-gfp* gene on the cell membrane, and also two additional cellulase genes (Figure 6). The presence of cellodextrin transporter might have helped the uptake of these smaller cellulose fragments in KR7 in the first three days of fermentation. When cellulases were secreted for cellulose digestion, the extra EglA and CBHII expressed in KR7 might have contributed to a higher cellulolytic efficiency than KR5 in the second and the third day of fermentation. The crystal avicel might have been gradually hydrolyzed by the synergistic cellulolytic enzyme reaction in the fourth day and the fifth day in KR7 (Figure 6). Although it was difficult to quantify the amount of substrate being degraded during fermentation with the saturated substrate level of 10% avicel, our study demonstrated that assembling an enzyme cocktail in an engineered yeast can improve the cellulosic ethanol productivity.

In the PGASO concept, each gene cassette contains the gene sequence linked, at the 5’ end, to a promoter sequence, and a sequence at the 3’ end of the gene cassette is identical to the 5’ end of the adjacent cassette. The promoter sequence in a gene cassette should be different from those of all other gene cassettes and the accuracy of gene cassette assembly was based on site-specific homologous recombination. In our previous study, the KR5 strain was derived from the transformation of five gene cassettes with a total length of ~15 Kb, and the procedure had 63% accuracy with the predesigned order assembly [24]. In this study, the KR7 strain was derived from the transformation of seven gene cassettes with a total length of ~22 Kb and the accuracy was decreased to 3%. Moreover, the quantitative PCR data indicated that more copies of the *cbhI, eglA, and npabgs* gene cassettes were inserted than the other gene cassettes, probably via the non-homologous end-joining (NHEJ) pathway [33]. NHEJ might cause an unanticipated result in genetic engineering. For example, the BGL activity in the supernatant of KR7 was 2.5-fold lower than KR5 (Figure 5B), which might be due to the 2-fold lower copies of the *npabgs* gene inserted in the KR7 genome [24]. Since NHEJ is a fairly common phenomenon in *Kluvyveromyces marxianus*, random gene insertion and screening for activity might be a good strategy for host engineering [23]. However, the random gene insertion approach is not convenient for transforming multiple genes into a host with desired gene copy numbers, and it is difficult to change the relative expression levels of different genes after they had been transformed. To increase the transformation accuracy of PGASO and to reduce the chance of an unanticipated result, we will try to reduce the NHEJ effect in KY3.

With *K. marxianus* KY3 as the host, the PGASO method has a high potential for practical applications. The fast growth rate and several other traits of KY3 make it desirable as a cell factory for commercial enzyme production. However, due to the saturated substrate level of 10% avicel, it was difficult to quantify the amount of substrate being degraded during the time course of fermentation of KR7. We measured the ethanol concentration at time zero of each culture in YP medium with avicel, and detected no significant ethanol residue. Also, a negative control was conducted by culturing KY3 in YP medium without avicel using OD 0.1 inoculums and OD 20 inoculums, and 0 g/l (OD 3) and roughly 0.3 g/l ethanol equivalent were detected after 5 days of culturing (data not shown). The ethanol equivalents and cell growth of KY3 might have been produced from the alternative metabolic pathway from peptone and amino acid in YP medium. Thus, our measurement of ethanol production was not accurate, but the data did show that KR7 could indeed convert avicel to ethanol, whereas KY3 and KR5 could not. Although this implies that <2% of the avicel was converted to ethanol under the current condition, it can be improved by elevating the reaction temperature or by other strategies. A better CBP fermentation parameter, such as SSR (solid-to-solution ratio), ORP (oxidation-reduction potential), and HRT (hydraulic retention time) will be considered to monitor the cellulose conversion rate in the future. Furthermore, to engineer *K. marxianus* KY3 to be a better CBP host for ethanol production, a number of issues still need to be resolved, such as expanding the variety of cocktail enzymes, increasing the number of gene cassettes, improving the pentose assimilating ability, decreasing the ethanol consumption rate and employing the cell surface engineering technology [24,34]. However, from the engineering point of view, this cellulolytic efficiency of CBP is not as “efficiently” as SHF (separate hydrolysis and fermentation) and SSF (simultaneous saccharification and fermentation), but is more economical in the enzymes used and operational cost. Although KR7 can convert cellulose to ethanol in one step and has many potential characters as a CBP strain, it is not good enough for industry ethanol production. Thus, further improvement is being pursued. Increasing the knowledge of the biology and the genome of *K. marxianus* will facilitate its applications in industry [13,35,36].

## Conclusion

Many fungi cannot produce a large amount of ethanol, but possess specialized enzymes that can efficiently degrade lignocellulosic materials. Using cellulase cocktail assays, we formulated a fungal cellulase cocktail with synergitic effects for cellulose digestion. Seven gene cassettes, including five cellulase genes, one cellodextrin transporter gene and a selection marker gene, were simultaneously introduced and expressed in KR7. KR7 could convert crystalline cellulose into ethanol. Our study demonstrates the potential of combining a cocktail formulation and a synthetic biology technique to develop a designer yeast host for the application in cellulosic ethanol industry.

## Methods

### Multiple-gene cassette construction

To investigate the activity of fungal cellulases, several cellulase genes were cloned and transformed into the *K. marxianus* KY3 host for enzyme assay. The genes for *cbhI* (Accession No. AY368686.1), *egIII* (Accession No. M19373.1), eglA (Accession No. XM_001397945.2), and npabgs (Accession No. JQ081958.1) were cloned from *T. reesei*, *A. niger*, and *Neo. patriciarum*. The template mRNA was purified using RNeasy mini kits (Qiagen, Chatsworth, CA) from the fungal cells, which were cultured under different conditions. *A. niger* CBS 122.49 and *T. reesei* ATCC 13631 were cultured with medium containing 2% malt extract and 2% cellobiose at 24°C with 150 rpm shaking for 3 days. *N. crassa* ATCC 14692 was cultured with NEUROSPORA MINIMAL MEDIUM (DIFCO 0817) plus 2% cellobiose at 24°C with 150 rpm shaking for 3 days. The buffalo rumen fungus *Neo. patriciarum* W5 was maintained in a rumen fluid-containing medium supplemented with 0.5% rice straw as the carbon and was anaerobically grown at 39°C for 3 days. The cDNA synthesis was conducted using a reverse transcription kit (SuperScript™ II kit, Invitrogen) for gene cloning. The *cbhII* (Accession No. on pending, submission ID: 1587754) gene was chemically synthesized by GeneScript Inc (Piscataway, NJ, USA) and was optimized for *K. marxianus* expression. Full length cDNA fragments for each gene were cloned into the pKlac2-kan vector, which was modified from the pKlac2 vector (*K. lactis* Protein Expression Kit, New England Biolabs), and transformed into *K. marxianus* KY3. These engineered yeast strains were cultured for investigating the cellulase synergistic reactions.

The engineered yeast KR7 was obtained via application of PGASO to assemble seven gene cassettes in a predesignated order as illustrated in Figure 3. In the first gene cassette, the *kanMX* gene and the *Lac*4 promoter fragment from pKlac2 vector were amplified and assembled into a fragment with the Lac4-KanMX primer pairs. The coding regions of the second to fifth gene cassettes, the *egIII*, *cbhI*, *eglA* and *cbhII* gene, were amplified and assembled with promoters including ScGapDH, KlADHI, ScPGK and KlPGK regions, respectively, by the designed primer pairs via fusion PCR. The sixth gene cassette with the gene cassette of *cdtI* (Accession No. XM_958708.1) was amplified from *N. crassa*, and then generated using the KlADHI-CDT-GFP primer pairs. The gene for the CDT-GFP fusion protein was constructed according to the report of expressing the CDT gene from *N. crassa* in yeast [13]. The GFP gene was fused at the C-terminal of the CDT gene without linker by primers NdeI-CDTI-F, CDTI-GFP-R, CDTI-GFP-F, EcoRI-GFP-R, and EcoRI-CDTI-R. The seventh gene cassette with the *npabgs* gene and a ScADHI promoter were respectively amplified and constructed using the ScADHI-NpaBGS primer pairs. Consecutive gene cassettes containing overlapping 55 bp regions on the border were used for recombinatorial gene assembly to designate as first to seventh of the gene cassettes. The primer pairs used in this study are listed in Additional file 1. The engineered *K. marxianus* yeast strains used in this study are listed in Table 1.

**Table 1:** **The engineered*K. marxianus*yeast strains used in this study**

**Strain name**	**Selection marker**	**Description**
KY3		KY3 wild type
KY3-NpaBGS	amds	KY3 transformed with the NpaBGS-pKlac2 vector
KY3-NpaBGS-CDT	Kan, amds	KY3 co-transformed with the NpaBGS-pKlac2 and CDT-GFP-pKlac2 vectors
KR5	Kan	KY3 co-transformed with the *kanMX, cbhI, egIII, gfp,* and *npabgs* genes
KR7	Kan	KY3 co-transformed with the *cbhII, cbhI, egIII, eglA, kanMX, cdtI-gfp,* and *npabgs* genes

### Yeast transformation and clone screening

The KY3 cells were incubated in 5 ml YPD medium (1% BactoDifco-Yeast Extract, 2% BactoDifco-Peptone, 2% Merck-D(+)-Glucose) at 30°C with shaking at 200 rpm for 16 hr. To express heterologous genes in KY3, we followed the transformation method for *K. marxianus*[24]. The target DNA fragments in 10 μl volume with an equal molar ratio of each fragment were mixed with 40 μl of competent cells. The electroporation was performed (1.0 kV, 400 Ω, and 25 μF capacitance) using a BioRad system (GenePluserXcell TM, Bio-Rad, Hercules, CA) with an aluminum cuvette (2 mm). The cells were spread onto YPG plates (1% BactoDifco-Yeast Extract, 2% BactoDifco-Peptone, and 2% Merck-galactose) containing G418 (200 μg/mL). To confirm the presence of each fragment, each isolated colony was digested in QucikExtract™ DNA Extraction Solution (EPICENTRE, Madison, Wisconsin) to remove yeast cell wall and was then examined by PCR with gene specific checking primers [see Additional file 1]. Moreover, to verify that these gene cassettes were inserted into the correct position of the KR7 genome and assembled in the correct order, specific internal primer pairs of gaps of each cassette were designed and confirmed by PCR [see Additional file 1]. These clones were further examined under a bright field microscope with phase contrast and fluorescence with a GFP filter, and photographed by a confocal microscope and single molecule detection system (Leica TCS-SP5-MP-SMD, Germany).

### Quantitative PCR analysis

The yeast cells were cultured in YPD medium at 30°C with shaking at 200 rpm for 16 hr. The genomic DNA was purified from yeast cells using a DNA Isolation Kit III (DNA Isolation Kit III, Roche). The template mRNA was purified from yeast cells using RNeasy mini kits (Qiagen, Chatsworth, CA). The cDNA synthesis was conducted using a reverse transcription kit (SuperScript™ II kit, Invitrogen). The relative quantification of each gene was carried out via the Universal Probe Library Set (LightCycler® 480 Probes Master, Roche) with a specific primer pair (the amplicon size was 100 to 150 bp) on a LightCycler (LightCycler 480, Roche), following the protocol of the manufacturer. Alg9 was used as a reference gene for quantitative PCR analysis. Standard curves were generated for each primer pair to estimate their amplification efficiency using the LightCycler software (LightCycler 480, Roche), and the quantitative PCR data were accordingly adjusted for use in subsequent analysis. The theoretical amplification efficiency is 2.0, and the actual amplification efficiencies of the different primer pairs were 1.97 (*cdtI-gfp*), 1.89 (*npabgs*), 1.93 (alg9), 1.94 (*egIII*), 1.93 (*eglA*), 2.0 (*cbhII*) 1.98 (*cbhI*), and 1.98 (*kanMX*).

### Quantitative assays of enzyme activity

To quantify the secreted cellulase activities, the supernatants of KY3, KR5, and KR7 were harvested and concentrated 100-fold for analysis. A total of 50 ml of culture supernatants were filtered through a 0.2-mm membrane (Sartorius, Goettingen, Germany), and then condensed using Vivaspin 20 (10-kDa cutoff) (GE Healthcare) at 4°C. Several enzymes, including Celluclast® 1.5 L (0.7 U/mg, Sigma aldrich C2730, USA), EGIII (64 U/mg, catalogue number E-CELTE, Megazyme International Ireland, Ireland), EglA (58.4 U/mg, Megazyme International Ireland, Ireland), CBHI (0.1 U/mg, catalogue number E-CBHI, Megazyme International Ireland, Ireland), purified CBHII (expression by KY3, 6.5 U/mg) and purified NpaBGS (expression by Phichia, 34.5 U/mg), were employed for enzyme cocktail assays. The same amount (110 μg) of crude extra-cellular enzymes collected from engineered yeast cultures and the same amount of each enzyme (15 μg) were used in several glucanase activity assays with different test substrates in a 1 ml test tube at 40°C. The same amount of each enzyme (7.5 μg) was used in the double enzyme assay. The protein concentration was determined by the Bradford method with a bovine serum albumin (BSA) standards.

Purification of NpaBGS from the Pichia-NpaBGS strain was performed according to the procedures described in Chen et al. [9]. The steps were as follows: The *Pichia* broth (4 L) was percolated through filter paper (Toyo Roshi Kaisha, Japan) and concentrated with a stirred ultrafiltration cell (model 8400; Millipore Corp.) equipped with a PM 10 membrane (Millipore Corp., USA) under the nitrogen pressure of 4.0 kg. f/cm2 and dialyzed against 20 mM sodium acetate buffer (pH 5.0). The extracted enzyme was condensed by precipitation at increasing concentrations of ammonium sulfate (0–30%, 30–50%, and 50–70%) at 5°C. The fraction contained a better activity and the amount of enzyme was found at 50–70% ammonium sulfate precipitation. The resulting precipitates were collected by cold centrifugation, dissolved in distilled water and dialyzed (0.1 M phosphate buffer, pH 6.0, 48 h, 5°C) to remove excessive salt. The protein (30 mL) was then loaded onto a Toyopearl DEAE-650 S (Tosoh, Japan) column (2.0 × 20 cm) and eluted with a step gradient of 0, 200, 300, 400, and 500 mM of NaCl in a volume of 1,000 mL. The fractions showing cellulolytic activity were pooled and concentrated by ultrafiltration, then dialyzed against 50 mM sodium acetate (pH 5.0) containing 0.15 M NaCl. The dialyzed sample (4 mL) was applied to a Sephacryl 300-S HR (GE Healthcare Bio-Sciences AB) column (1.6 × 60 cm) and eluted with the same buffer at a flow rate of 0.5 ml/min. The active fractions were concentrated by ultrafiltration and dialyzed against 20 mM Tris–HCl buffer (pH 8.0) containing 1.5 M (NH4)2SO4. The dialyzed enzyme solution (2.0 mL) was then loaded onto a Resource PHE (Amersham Biosciences, USA) column (1.0 × 1.0 mL) equilibrated with the same buffer containing 1.5 M (NH4) 2SO4. The active fractions were eluted with a decreasing linear gradient from 1.5 to 0 M of (NH_4_)2SO_4_ in the buffer at a flow rate of 1.0 mL/min. To purify CBHII, the supernatant of KY3-CBHII was harvested and concentrated 100-fold for analysis. A total of 50 ml of culture supernatant was filtered through a 0.2-mm membrane, and then condensed using Vivaspin 20 (10-kDa cutoff) at 4°C.

The CBH and FPA activity was assayed by adding 100 ml of supernatants to 900 ml buffer solution containing 50 mM sodium acetate (pH 5) and 0.4% phosphoric acid-swollen cellulose (PASC) or 50 mg Whatman filterpaper #1, respectively. PASC was prepared by the following steps: A cellulose slurry of 30 g Sigmacell 50 (Sigma-Aldrich Co., St. Louis, MO) in water was slowly added to 2.5 L of concentrated 85% phosphoric acid at 4°C. The suspension was stirred at 4°C for 1 hour before precipitating in 10 L of water. The precipitated cellulose was washed extensively with water until the final pH equilibrated at pH 5. The PASC was autoclaved and stored at room temperature. The assay tubes were incubated at 40°C for 24 hrs. After the hydrolysis reaction, the amount of glucose was measured by using the glucose (HK) assay kit (Sigma-Aldrich, USA) to determine the number of glucose equivalents. To formulate an efficient cellulase cocktail for CBP, the filter paper assay in this study used the crude extra-cellular enzyme samples and reacted in a 1 ml testing tube at 40°C for 8 hours. The cellulase activity was defined by the FPA assay, and one unit of FPA is defined as 1 mmol reducing sugar released from filter paper in 1 minute. The EG activity was assayed by mixing 40 μl of supernatants with 60 μl of buffer solution (2% AZO-CM-Cellulose (Dye-CMC), 50 mM sodium acetate, pH 5) at 40°C, 30 mins. The enzyme activity was measured by optical absorptive intensity reader (SpectraMax M2, MDS) with OD 590 nm. The quantitative assay of the BGL activity was similar to the above method at 40°C, 10 mins, but 50 mM p-nitrophenyl-D-glucose (pNPG) was used as the substrate and detection was done via absorbance under absorption 410 nm. The protein concentration was determined by the Bradford method with a bovine serum albumin (BSA) standards.

### The cellulosic ethanol conversion assay

The cellodextrin substrate used in this study was a commercial formula product, i.e., a low viscosity barley beta-glucan (Megazyme International Ireland, Ireland, Ireland). The cellodextrin substrate was used as an index for improving cellulose hydrolyte assimilating ability, but the efficiency of cellulose conversion should be estimated by raw cellulose substrate, such as avicel. The transformed yeast cells were grown in the substrate concentration of 10% avicel (Avicel® PH-101, Sigma-Aldrich Co. LLC, USA) as the carbon source, for the ethanol conversion assay. After cultivation in the YPD medium for 24 h at 30°C, KY3, KR5, and KR7 cells were harvested for subsequent high cell density inoculum. These cells were washed by ddH_2_O three times at 4°C and the initial inoculated cell density for each sample had an OD of 20 at a wavelength of 600 nm using a spectrophotometer (Ultrospec 2100 pro; Amersham Bioscience). The dry avicel powder was separately autoclaved from the YP medium via a vertical autoclave in a 15 ml serum tube and supplied by subjecting to 1.2 atm (kg/cm2) high pressure saturated steam at 121°C for around 20 minutes. The semi-anaerobic batch culturing was conducted in a 15 ml serum tube with 10 ml of medium with 5 ml of air in the bottle at 40°C, 250 rpm rolling. The production of ethanol was analyzed by gas chromatography (Shimazdu, GC-14, Japan) with a flame ionization detector (FID) and a stainless steel column (80/120 Carbopack B/6.6% Carbowax, 2 m x 2 mm), with nitrogen as mobile gas. The running condition included heating of the column from 80 to 150°C at a ramp rate of 4°C per min, an injection temperature of 180°C, and a detection temperature of 250°C. Each fermentation experiment and the subsequent analysis were repeated three times.

## Abbreviations

CBP: Consolidated bioprocessing;PASC: Phosphoric acid-swollen cellulose;Dye-CMC: AZO-CM-Cellulose;pNPG: p-nitrophenyl-D-glucose;FPA: Filter paper activity;CBH: Cellobiohydrolases;EG: Endo-β-1,4-glucanases;BGL: β-glucosidases

## Competing interests

The authors declare that they have no competing interests.

## Authors’ contributions

J-JC designed experiments and drafted the manuscript. F-JH, C-YH, Y-CW, and Y-HH carried out the experiments, and analyzed the data. W-HL, C-CH and M-CS supervised the study and revised the manuscript. All authors read and approved the final manuscript.

## Supplementary Material

Additional file 1 The primer pairs used in the KR7 construction. Click here for file
