# Supplementary material for: Assembling a cellulase cocktail and a cellodextrin transporter into a yeast host for CBP ethanol production
Source: Biotechnol Biofuels. 2013 Feb 4;6:19. doi: 10.1186/1754-6834-6-19 (PMC3599373; doi:10.1186/1754-6834-6-19)
Supplement: Additional file 1 — The primer pairs used in the KR7 construction. [file 1754-6834-6-19-S1.docx]

**Additional file 1. The primer pairs used in the KR7 construction.**

| **Primer name** | | **Sequence** |
| --- | --- | --- |
| **Cassette construction** | |  |
| Lac4-KanMx | Rainbow 1 | 5’-ccgcggggatcgactcataaaatag-3’ |
|  | Rainbow 2 | 5’-CTACTATTAATTATTTACGTATTCTTTGAAATGGCAGTATTGATAATGATAAACTTAT-3’  ACAACATCGAAGAAGAGTCT-3’ |
| ScGapDH-EgIII | Rainbow 3 | 5’-AGTTTATCATTATCAATACTGCCAT-3’ |
|  | Rainbow 4 | 5’-GGACTCCAGCTTTTCCATTTGCCTTCGCGCTTGCCTGTACGGTCGTTACCATACTTGG  CGGAAAAAATTCATTTGTAA-3’ |
| KlGapDH-CBHI | Rainbow 5 | 5’-AGTATGGTAACGACCGTACAGGCAA-3’ |
|  | Rainbow 6 | 5’-AATCGATTTACAGAAACTTGCACACTAAAAATACACAACTAAAAGCAATTACAGTT  GGCGGAAAAAATTCATTTGTAA-3’ |
| SCPGK-EgI | Rainbow 7 | 5’- ACTGTAATTGCTTTTAGTTGTGTAT-3’ |
|  | AFEgI_ScPGK_R | 5’-AATATAGTAGAGAATTTCATttttttTGTTTTATATTTGT-3’ |
|  | ScPGK_AFEgI_F | 5’-ACAAATATAAAACAaaaaaaATGAAATTCTCTACTATATT-3’ |
|  | ScTTPGK_EgI_R | 5’-CGATTTCAATTCAATTCAATTTACTCAGCAACGCACTGGT-3’ |
|  | EgI_ScTTPGK_F | 5’-ACCAGTGCGTTGCTGAGTAAATTGAATTGAATTGAAATCG-3’ |
|  | Rainbow 8 | 5’-CATATACCTTTGATACCATAAAAACAAGCAAATATTCTTACTTCAAACACACCCGA  AGCTTTTTCGAAACGCAGAATTTT-3’ |
| KlPGK-CBHII | Rainbow 9 | 5’-CGGGTGTGTTTGAAGTAAGAATATT -3’ |
|  | AFCBHII_KlPGK_R | 5’-AATATAGTAGAGAATTTCATTTTTTTATTAATTCTTGATC-3’ |
|  | KlPGK_AFCBHII_F | 5’-GATCAAGAATTAATAAAAAAATGAAATTCTCTACTATATT-3’ |
|  | ScTTPGK_CBHII_R | 5’-CGATTTCAATTCAATTCAATTTAGAAGGCTGGGTTAGCGT-3’ |
|  | CBHII_ScTTPGK_F | 5’-ACGCTAACCCAGCCTTCTAAATTGAATTGAATTGAAATCG-3’ |
|  | Rainbow 10 | 5’-AGGTAAGTATGGTAACGACCGTACAGGCAAGCGCGAAGGCAAATGGAAAAGCTG  GAAGCTTTTTCGAAACGCAGAATTTT-3’ |
| KlADHI-CDTI-GFP | Rainbow 11 | 5’-CCAGCTTTTCCATTTGCCTTCGCGCTTGCC-3’ |
|  | CG_KlPADHI_R | 5’- CGTCATGGGAGCCGTGAGACGACATTTTATCTTTTTTTAGTATAGAGTTT-3’ |
|  | KlPADHI_CG_F | 5'-AAACTCTATACTAAAAAAAGATAAAAtgtcgtctcacggctcccatgacg-3 |
|  | ScTTGap_CG_R | 5’- AATGCAAGAT TTAAAGTAAA TTCACTTACT TGTACAGCTC GTCCATGCCG-3’ |
|  | CG_ScTTGap_F | 5’-CGGCATGGACGAGCTGTACAAGTAAGTGAATTTACTTTAAATCTTGCATT-3’ |
|  | Rainbow 12 | 5’-GGAATCCCGATGTATGGGTTTGGTTGCCAGAAAAGAGGAAGTCCATATTGTACACT  GGCGGAAAAAATTCATTTGTAA-3’ |
|  | NdeI-CDTI-F | 5’-ACTCATATGAtgtcgtctcacggctcccatgacg-3’ |
|  | CDTI-GFP-R | 5’-AGCTCCTCGCCCTTGCTCACagcaacgatagcttcggaca-3’ |
|  | CDTI-GFP-F | 5’-tgtccgaagctatcgttgctGTGAGCAAGGGCGAGGAGCT-3’ |
|  | EcoRI-GFP-R | 5’-TATGAATTCTTACTTGTACAGCTCGTCCATGCCG-3’ |
|  | EcoRI-CDTI-R | 5’-TATGAATTCctaagcaacgatagcttcggacaCAT-3’ |
| ScADHI-NpaBG | Rainbow 13 | 5’-GTGTACAATATGGACTTCCTCTTTTC-3’ |
| ScADHI-NpaBG | Rainbow 14 | 5’-gaaatttaggaattttaaacttg-3’ |
| **Checking primer** | |  |
| Kan | Kan-BglII F | 5’-AAAAAGATCTGCCACCATGGGTAAGGAAAAGACTC-3’ |
|  | Kan-XbaI R | 5’-AAAAATCTAGATTAGAAAAACTCATCGAGCAT-3’ |
| EgIII | EgIII-88 F | 5’-GAGGTATTGGTTGGAGCGGACCTAC-3’ |
|  | EgIII-1060 R | 5’-TCTTGTATGCAGGACTGAACGTTGC-3’ |
| CBHI | CBHI-192 F | 5’-ACGAACAGCAGCACGAACTGCTACGATGGCAACACTT-3’ |
|  | CBHI-1260 R | 5’-GAGACTGAGATTCGACCTGAGCAGG-3’ |
| EgI | EgI F | 5’-ATGAAATTCTCTACTATATTAGC-3’ |
|  | EgI R | 5’-TTACTCAGCAACGCACTGGTAGT-3’ |
| CBHII  CDTI-GFP | CBHII F | 5’-TGAAATTCTCTACTATATTAGCCGC-3’ |
|  | CBHII-1029 R | 5’-AATGAAGTGAGCGTCGAAACCTTGG-3’ |
|  | NdeI-CDTI F | 5’-ACTCATATGAtgtcgtctcacggctcccatgacg-3’ |
|  | EcoRI-CDTI R | 5’-TATGAATTCctaagcaacgatagcttcggacaCAT-3’ |
| NpaBGS | NpaBGS-1422-F | 5’-TCCAGGTCCAGTTAATGTTCCATTC-3’ |
|  | NpaBGS-SmaI-R | 5’-TATCCCGGGTTAGTAAAGTTTGTAAGC-3’ |
| **Internal primer** | |  |
| amplicon 1 L-K | Lac4-Primer1 | 5’-ACACACGTAAACGCGCTCGGT-3’ |
|  | Kan-126R | 5’-TACAATCGATAGATTGTCGCACCTG-3’ |
| amplicon K-EIII | Kan-673F | 5’-CAGGATCTTGCCATCCTATGGAACT-3’ |
|  | EgIII-528R | 5’-TACTTGGAAATGCTCGTGGAATCAA-3’ |
| amplicon EIII-CI | EgIII-1084F | 5’-GACATGTGCCAGCAAATCCAATATC-3’ |
|  | CBHI-218R | 5’-AAGTGTTGCCATCGTAGCAGTTCGT-3’ |
| amplicon CI-EI | CBHI-585F | 5’-CGATCTGAAGTTCATCAATGGCCAG-3’ |
|  | EGI-91-R | 5’-AGCCAGAGTCACGCTTCTTCATCTG-3’ |
| amplicon EI-CII | EGI-1071-F | 5’-TACCTCCACTGCTTCCACCACCACT-3’ |
|  | CBHII-1029-R | 5’-AATGAAGTGAGCGTCGAAACCTTGG-3’ |
| amplicon CII-G | CBHII-1144-F | 5’-CAACCACTGACACTGGTGATGCCT-3’ |
|  | CDT-55-R | 5’-GAGTAGCAAGATGCTTCTCGGTGCT-3’ |
| amplicon G-N | GFP-492F | 5’-GAACTTCAAGATCCGCCACAACATC-3’ |
|  | NpaBGS-403R | 5’-CACATTCACCAACATAGAATGGATC-3’ |
| amplicon N-L | 196-1422F | 5’-TCCAGGTCCAGTTAATGTTCCATTC-3’ |
|  | Lac4-3'-436-R | 5’-ACTCTACATGCGACTTGGAAGGC-3’ |
| **UPL system QPCR primer** | |  |
| Kan | Kan-UPL#144F | 5’- agactaaactggctgacggaat-3’ |
|  | Kan-UPL#144R | 5’- catcaggagtacggataaaatgc-3’ |
| EgIII | EgIII-UPL#77F | 5’- tggctccgacagaacaatc -3’ |
|  | EgIII-UPL#77R | 5’- gtcttgtatgcaggactgaacg -3’ |
| CBHI | CBHI-UPL#77F | 5’- acatcaagttcggacccatt-3’ |
|  | CBHI-UPL#77R | 5’- ggtaggtccgggagagctt-3’ |
| EgI | EgI-UPL #75 F: | 5’- ggcgtcaactccgtgtgt-3’ |
|  | EgI-UPL# 75 R: | 5’- aggaagcaccaacccagac-3’ |
| CBHII | CbhII-UPL #60 F: | 5’- cgcttccgtttacaagaacg-3’ |
|  | CbhII-UPL #60 R: | 5’- aacgttggtagccaaacctc-3’ |
| GFP | GFP-UPL#148F | 5’- tctatatcatggccgacaagc-3’ |
|  | GFP-UPL#148R | 5’- gttgtggcggatcttgaagt-3’ |
| NpaBGS | NpaBGS-UPL#150F | 5’- gaagctgtaatggaagaagatgg-3’ |
|  | NpaBGS-UPL#150R | 5’- ctgggaatgaaaggaaaatcat-3’ |
| Alg9 | Alg9-UPL#151F | 5’- gtgggtctataccacgtctcatc-3’ |
|  | Alg9-UPL#151R | 5’- tccaaatataacgaatttaagcaactt-3’ |
| Actin | Actin-UPL #9F | 5’- GCGTAGATTGGAACAACGTG-3’ |
|  | Actin-UPL #9R | 5’- AGAACTACCGGTATTGTGTTGGA-3’ |
